# Mobile applications in radiology: own study based on polish data

**DOI:** 10.1038/s41598-023-46272-z

**Published:** 2023-11-16

**Authors:** Jakub Kufel, Katarzyna Bargieł, Maciej Koźlik, Wiktoria Bartnikowska, Michał Janik, Łukasz Czogalik, Piotr Dudek, Dariusz Krawczyk, Mikołaj Magiera, Maciej Cebula, Zbigniew Nawrat, Katarzyna Gruszczyńska

**Affiliations:** 1 Department of Biophysics, Faculty of Medical Sciences in Zabrze, Medical University of Silesia, 41-808 Zabrze, Poland; 2 Department of Biophysics, Faculty of Medical Sciences in Zabrze, Medical University of Silesia, Jordana 19, 41-808 Zabrze, Poland; 3 Division of Cardiology and Structural Heart Disease, Medical University of Silesia, 40-635 Katowice, Poland; 4 Individual Specialist Medical Practice Maciej Cebula, 40-752 Katowice, Poland; 5 Department of Radiodiagnostics, Invasive Radiology and Nuclear Medicine, Department of Radiology and Nuclear Medicine, School of Medicine in Katowice, Medical University of Silesia, Medyków 14, 40-752 Katowice, Poland

**Keywords:** Computational biology and bioinformatics, Health care, Medical research

## Abstract

As the number of smartphones increases, so does the number of medical apps. Medical mobile applications are widely used in many medical fields by both patients and doctors. However, there are still few approved mobile applications that can be used in the diagnostic-therapeutic process and radiological apps are affected as well. We conducted our research by classifying radiological applications from the Google Play® store into appropriate categories, according to our own qualification system developed by researchers for the purposes of this study. In addition, we also evaluated apps from the App Store®. The radiology application rating system we created has not been previously used in other articles. Out of 228 applications from the Google Play store, only 6 of them were classified as “A” category with the highest standard. Apps from the App Store (157) were not categorized due to the lack of download counts, which was necessary in our app-rating system. The vast majority of applications are for educational purposes and are not used in clinical practice. This is due to the need of obtaining special permits and certificates from relevant institutions in order to use them in medical practice. We recommend applications from the Google Play store that have been classified in the “A” category, evaluating them as the most valuable. App Store apps data is described and presented in the form of diagrams and tables.

## Introduction

The number of people who own smartphones is growing rapidly. In 2016, there were 3.7 billion smartphone users in the world, while the estimated number for 2022 was over 6.5 billion^1^. Simultaneously, the number of medical applications is growing, which has been especially noticeable over the last few years^2–4^. With the increasing number of medical applications, the value of the medical applications market is growing as well—in 2021 it was priced at USD 5.37 trillion, while by 2029 it’s estimated to be worth USD 38.47 trillion^5^. In a study conducted among smartphone users, almost half of the respondents (47.7%) used at least one health app that was related to wellness, fitness, prevention, medication, treatment or follow-up care^6^.

Constantly-growing mobile application market has also met the needs of radiologists and there are many applications related to diagnostic imaging. We can distinguish radiological image viewers (so-called Digital imaging and communications in medicine viewers—DICOM viewers), platforms for exchanging photos between doctors or different medical centers, instructions for correct patient positioning, guidelines of radiological scientific societies, applications for learning radiological anatomy, quizzes and databases of questions with radiological issues. Mobile radiological applications are also used in remote image interpretation, which is especially useful in places where there are no radiologists^7^. Unfortunately, applications that are intended for clinical usage are most often used for educational purposes due to the lack of appropriate certification. However, it has been shown that the interpretation of CT examinations on tablets or smartphones despite small screen resolution is possible and the assessment made with the help of a mobile device is similar to that made with the help of picture achieving and communication system (PACS) station^8,9^. Using mobile devices to evaluate diagnostic images is possible because they have enough computing power to quickly process full exams^7^. Radiology applications are also used to educate students and doctors^10^. What is more, radiological journals, out of concern for their readers, create mobile applications which facilitate access to their content^11^.

Other authors addressing the topic of comparing mobile solutions in radiology have taken a number of different strategies in selecting sources for collecting data for analysis. An article on the mobile medical apps market published on globenewswire.com presents a qualitative study and analysis of mobile medical apps in general. The report is economic in nature, so it serves primarily business and IT issues^5^. Chen et al. present a pair of tools for radiology education, with specific characteristics. The work has the character of an approximation of the capabilities of selected platforms and teaching methods. The review was inspired by the need to implement remote learning in the face of the COVID-19 pandemic^10^. Rahmani et al. looked at mobile apps used by radiology journals—only the Apple® App Store™ was searched for this purpose^11^. Kufel et al. created a systematic review of the scientific literature comparing mobile DICOM image viewers described in reports available in public medical databases (e.g., PubMed)^12^.

We conducted our own research aimed at evaluating mobile applications related to radiology, which are available at Google Play store and App Store, and then selected the best and most useful for users.

## Methodology

### Search strategy and selection criteria

The applications were selected by entering the term 'radiology' into polish version of Google Play store and App Store. Then, the necessary data was entered into a spreadsheet for all the applications found. Data concerned the origin of the application (Google Play, App Store), the number of downloads, the date of the last update, the presence of contact details of the application author and the application name. In addition, applications were classified in terms of use (educational/clinical use). The aspect of application presentation in a given store was also assessed (visual presentation of the application by means of videos, photos, presence of a website related to the application, description of the application). App Store does not present information about the number of application downloads, application's evaluation in the store and the contact details of application author.

Data on payment for the application (app price and in-app payments) was also obtained, however, this parameter was not included in the application evaluation.

### Data extraction and quality assessment

In September 2022, Google Play and the App Store were searched. Due to large discrepancies in access to data between these two stores it was not possible to evaluate these applications in the same way. Therefore, they were assessed independently using a separate methodology:

#### Google Play

##### Assessment of the visual presentation of the application (based on data from the store)

Scoring system used during the visual evaluation of the application:

- Presence of photo(s)—1 point—It allowed for an initial assessment of the application's functionality and familiarization with its capabilities.
- Presence of film(s)—1 point—It presented in a better way the capabilities of the software and the usefulness for a potential user.
- Presence of description—1 point—An application with a description is more credible, in particular a description of updates and improvements made, which gives the impression of a "not abandoned" application.
- The presence of a dedicated website—1 point—Website usually contains contact with the author enabling technical support, FAQ, a detailed description of the possibilities and functionalities of the application.

In total, the application could be assessed visually from 0 to 4 points.

##### Last update date

It may indicate the involvement of the application author in its creation, updating knowledge, improving operation and functionality, as well as correcting any errors. It is also the most reliable source of information about the validity of the contained data. To assess the “last update date”, date ranges were used, for which various point values from 0 to 5 points were awarded (Table 1).

**Table 1:** Points assignment system for the date of the last update.

From	To	Points
0	2012	0
2013	2014	1
2015	2016	2
2017	2018	3
2019	2020	4
2021	2022	5

##### Number of installations

The number of application installations was divided into numerical ranges, to which appropriate point values were assigned from 0 to 5 points. It was possible by rounding the number of downloads to full values in the Google Play Store (Table 2).

**Table 2:** Point assignment system for the number of installations.

From	To	Points
0	1000	0
1001	5000	1
5001	10,000	2
10,001	50,000	3
50,001	100,000	4
100,001		5

Finally, a formula was created that, based on all the components, awarded points to individual applications from the database and assigned them the appropriate category from A to F (Table 3).

1 $$ \Sigma {\text{ Ultimate points}} = \left( {\left( {{\text{X1}} + {\text{X2}} + {\text{X3}}} \right)/\left( {{14}} \right)} \right)*{1}00\% $$

**Table 3:** Category assignment system.

From	To	Category
0,00%	16,64%	F
16,65%	31,31%	E
33,32%	49,98%	D
49,99%	66,65%	C
66,66%	83,32%	B
83,33%	100,00%	A

14—the maximum number of points to be scored in the assessed categories.

#### App Store

##### Visual evaluation of the application (based on data from the store)

Unfortunately, due to the insufficient amount of available data, we were unable to perform a visual evaluation of the application at a level comparable to applications from Google Play store.

##### Last update date

It may suggest the degree of application developers’ involvement in its functioning, their care for the optimal, error-free operation of the program and the validity of knowledge or clinical data contained in the application.

##### Number of installations

Unfortunately, this stat cannot be obtained from the App Store. Therefore, it was decided to omit it from the work.

### Detailed inclusion and exclusion criteria

In order for a given application to be qualified for this study it had to meet all of the following criteria: application was the result of a search for “radiology” in the search engine in a mobile application store, it had clinical or educational use, it dealt with radiology.

In order for an application not to be qualified for this study it had to meet at least one of the following criteria: application did not deal with radiology, it was related to scientific publishing houses, conferences or was of an entertainment nature (was about body scanner games and such).

### Ethical approval

Not applicable. The scientific study we conducted was not a medical experiment. It was conducted without the involvement of humans or animals. Furthermore, no biological materials derived from humans or animals were used. Therefore neither medical nor personal information in any form was collected or collected from study participants.

## Google Play and App Store review results

### Google Play

**Figure 1:**
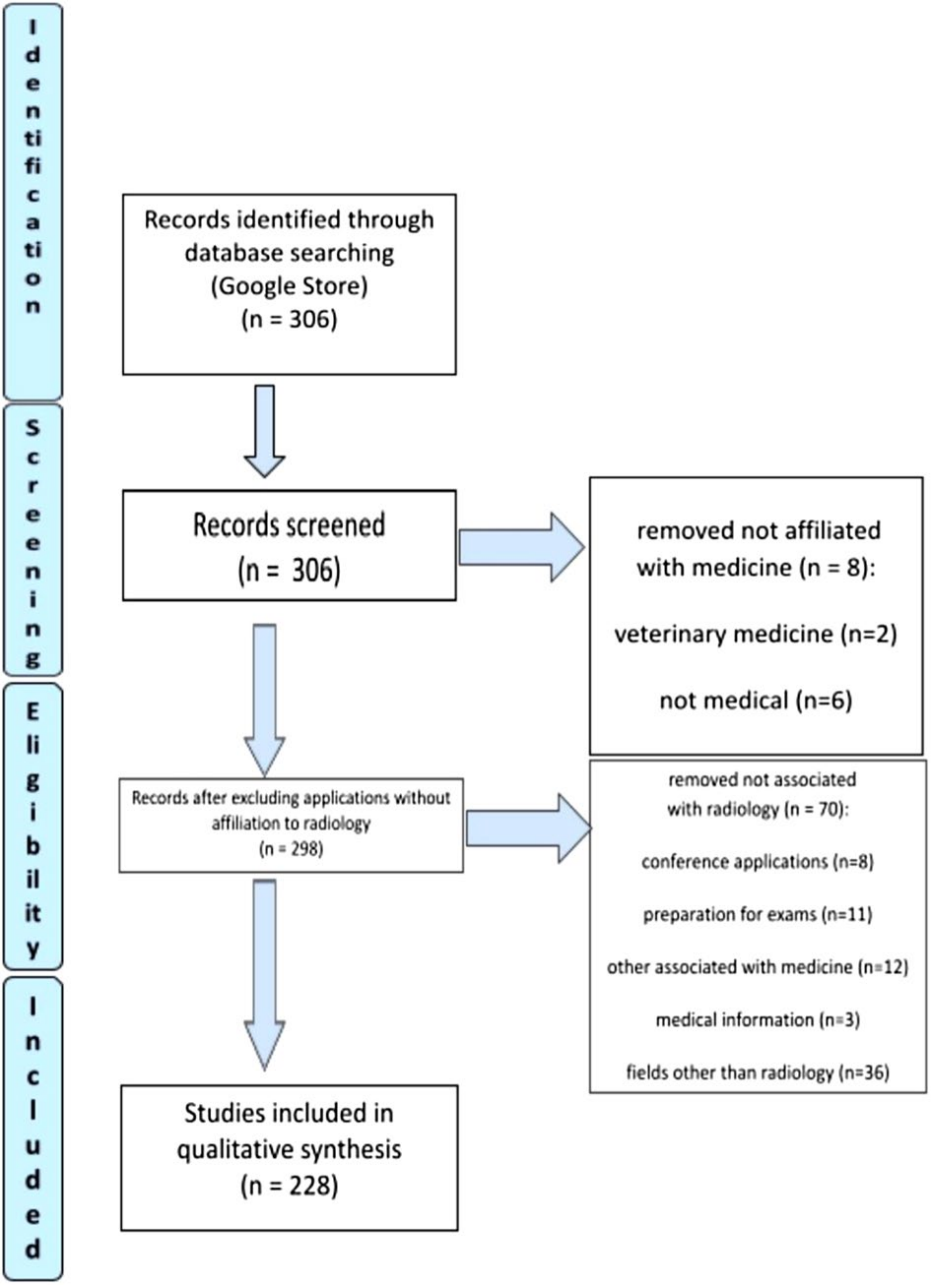
Google Play—flow diagram.

Initial results showed 306 records. 78 applications were excluded from the study with reasons. The remaining applications were added to the spreadsheet for further review of any information that is covered in detail in the methodology. Ultimately, 228 applications were included in further testing. Whole process is presented in the Flow Diagram 1 (Fig. 1).

**Figure 2:**
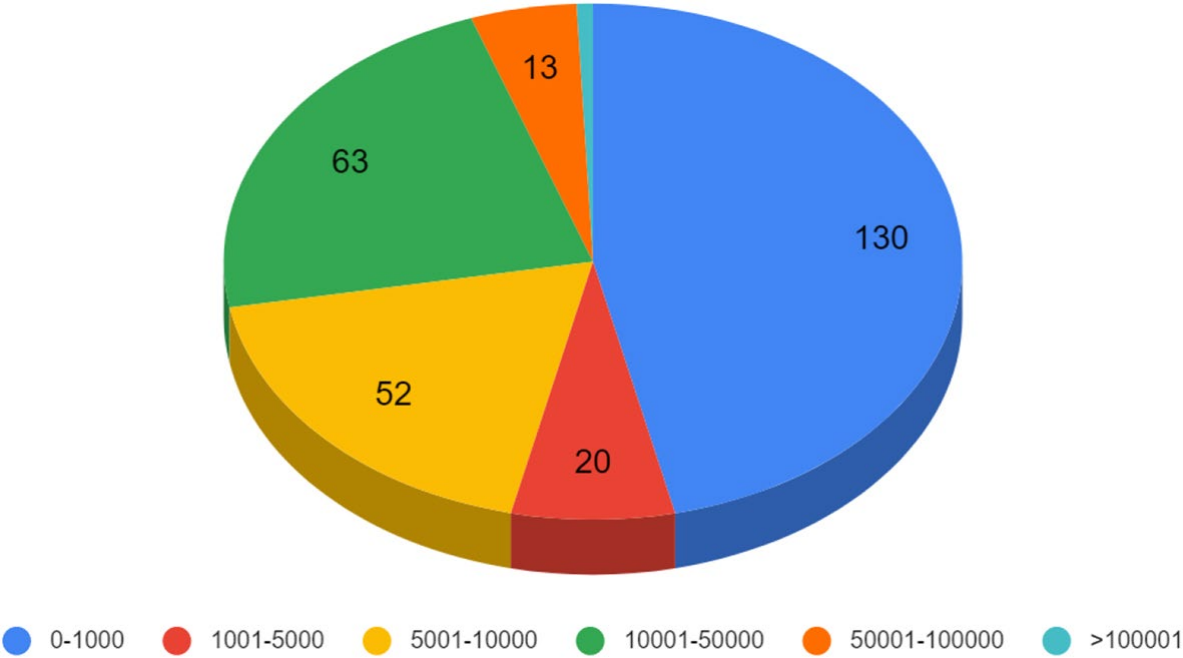
Number of Google Play app downloads in numerical ranges.

It can be seen that the largest group (130 applications) in terms of the number of downloads is the group from 0 to 1000 application downloads. The least numerous group is a group with > 100,001 application downloads, which includes only 2 applications (Fig. 2).

**Figure 3:**
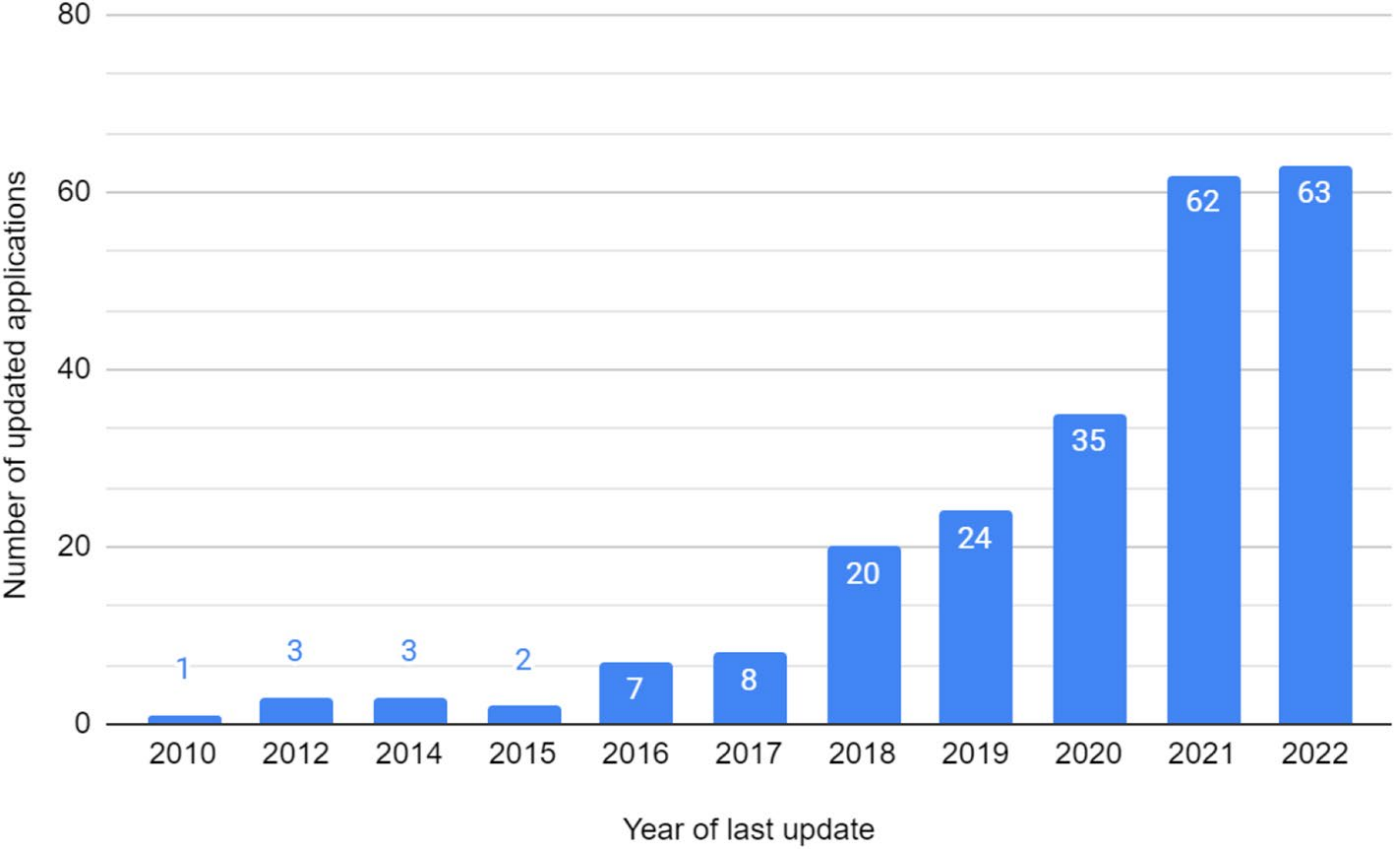
Number of applications that were last updated in a given year.

The number of applications updated in 2022 was 63, which shows that the most up-to-date applications account for 27.63% of all qualified applications from the Google Play store (Fig. 3).

Most of the applications qualified for the review were completely free (207 from 228). 5 of them were paid before downloading, while the remaining 16 applications were free to download with the possibility of making additional paid purchases through the application. The prices ranged from PLN 9.19 (EUR 1,99, USD 2,11) for the “Infection & Arthritis X-ray QZ Skeletal Radiology®” application to PLN 97.99 (EUR 21,26, USD 22,54) for “Navi Radiography Pro®”.

The largest group of all applications qualified for the study were educational applications (154). We also decided to distinguish between applications that may be clinically useful (59) and those that may be both educational and clinically used (15). It should be emphasized that none of the above-mentioned applications has the appropriate permission for use in clinical practice, therefore it can only be used as an advisory tool.

The letter “A” in the classification means applications with the best scores. They have been classified in this category because they represent the highest level of credibility, usefulness and functionality, which is also reflected in the correlation between the high number of votes and very high rating of users. The compatibility of photos, descriptions and videos (if any) is also evidenced by positive user comments, along with feedback from the application developers. The letters "B", "C", "D", and "E" represent successively worsening levels both in terms of visual, quantitative (in terms of the number of installations) and qualitative. The letter "E" denotes the applications that represent the lowest level. The number of the best applications (“A” category) was equal to the number of the worst (“E” category) and amounted to 6. 35 applications were qualified to the “B” category, 149 to “C” and 32 to “D” category.

From the Google Play Store apps included in this study, the 6 apps received “A” category (Table 4).

**Table 4:** Mobile applications with “A” category.

Name of the app	Description
MANUAL RADIOLOGY 3®	Third part of the radiology manual, containing thousands of files with radiological examinations in many projections to download. The application offers users the opportunity to use chat to exchange experiences and knowledge. It’s free but requires internet access. It is possible to return to a given image in offline mode
e-Anatomy®	Application based on the online atlas of human anatomy IMAIOS, which contains over sixteen thousand images in various projections, as well as radiographs, charts and illustrations. The app is available in 12 languages and has many functions to facilitate use, e.g. zooming in and out of the image or index search. It is intended for licensed medical professionals, i.e. physicians, radiology technicians and medical students. It’s costs 89.99 euro per year and buying a subscription also gives you access to the atlas on the IMAIOS website
The DroidRender—3D DICOM viewer®	Free viewer of radiological images in DICOM format, which supports educational institutions. It has a number of functions for image processing, such as free slicing for 2D and 3D view, the ability to obtain reconstructions or measuring
Basic Radiology for medical students®	Free application designed for medical students, containing basic radiology knowledge in the form of flashcards and multiple-choice questions, the purpose of which is to help prepare for Radiology Fellow of Royal College of Radiology (FRCR) exams. There are three levels of difficulty, numerous images, animations and illustrations as well as the ability to choose the topics that the user wants to repeat at any given time. Downloading the application also allows you to contact the tutor
RX—Posiciones Radiológicas®	Free application created for all people interested in radiology. It allows you to quickly and intuitively learn radiological positions so that the performed examinations are visually correct and more comfortable for the patient. Users can also view practical examples of X-ray images and the corresponding correct patient positions
Radiology Signs®	The only mobile application available in the world that collects all typical symptoms observed in various types of radiological examinations, such as classic X-ray, CT, MRI, ultrasound and Nuclear medicine examinations. In addition, it is completely free for users. It has a number of functions to facilitate searching for terms and contains a brief description of each symptom

### App Store

**Figure 4:**
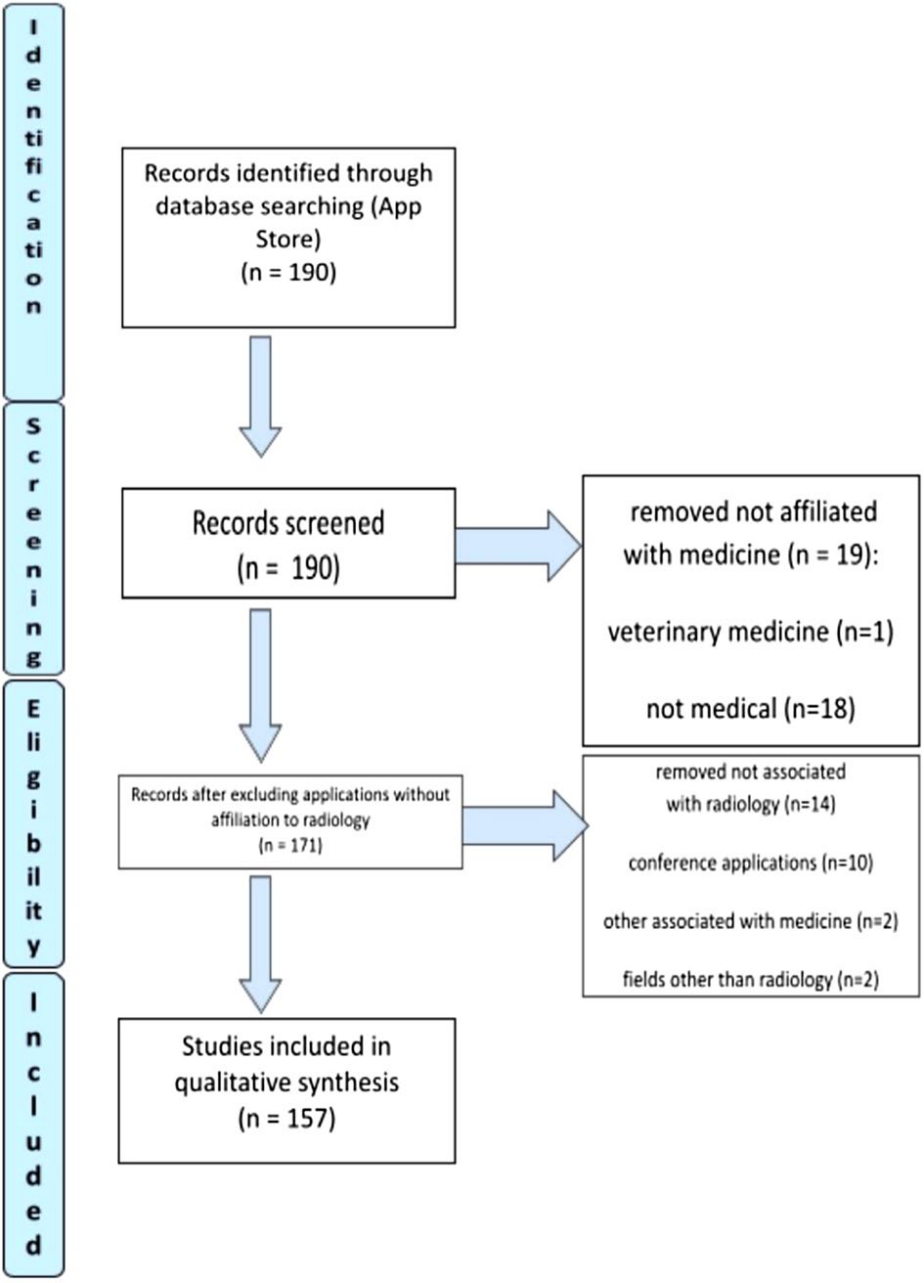
App Store—flow diagram.

In September 2022, an analysis of the store's offer was carried out and showed 190 results. 33 applications were excluded from the study with reasons. Remaining applications have been added to the spreadsheet for further review which is covered in detail in the methodology section. Ultimately, 157 applications were included in this study. Whole process is presented in the Flow Diagram 2 (Fig. 4).

Most of the apps qualified for the review were free (119). The number of paid applications was as numerous as those containing an additional paid application (19). Prices for paid applications ranged from PLN 4.99 for 'Thorax—thoracic radiology®' to PLN 529.99 for 'M3 TRR®'.

The largest group of qualified applications was the group containing applications for educational purposes (104), and 4 of them could also be used in medical practice. 49 applications were intended solely for diagnostics during daily clinical practice. It should be noted again that the presented applications do not have the necessary permissions to be officially and legally approved for use in clinical practice, so they can only be a helpful tool for both patients and clinicians.

**Figure 5:**
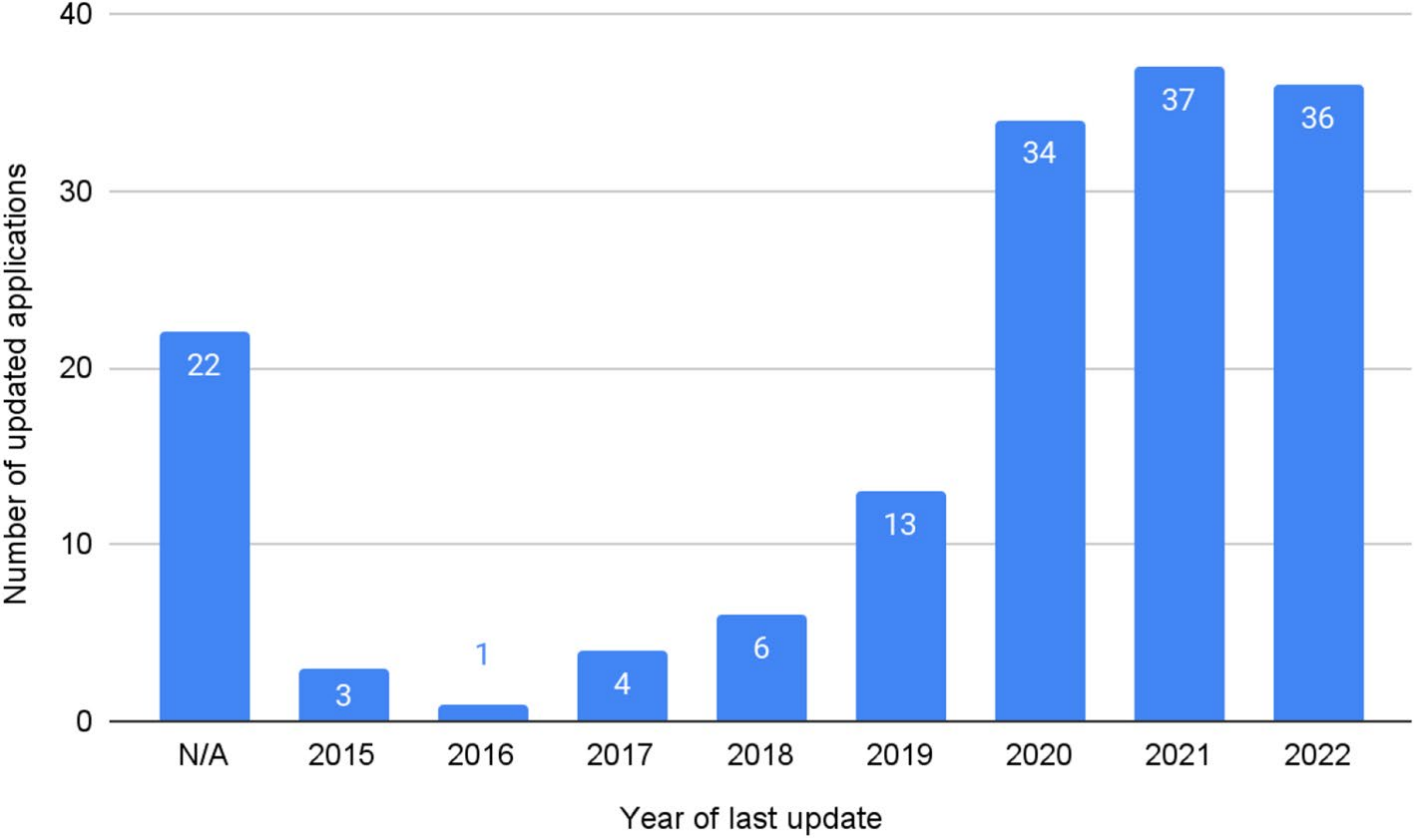
Number of applications that were last updated in a given year.

The number of applications updated in 2022 was 36, which shows that the most up-to-date offers account for 22.92% of all applications analyzed in this review (Fig. 5). It is worth mentioning that due to the partially lacking data on the date of the last update, we were unable to determine this parameter for 22 apps from the App Store.

## Discussion

Strictly medical mobile applications are not very popular among users and deserve more attention and recognition of their potential, both in the educational field and as a helping tool in everyday clinical practice. Their implementation in clinical fields improves both the efficiency and quality of health care systems and has a positive impact on the time of diagnosing^12^. The use of mobile devices together with appropriate applications enables the interpretation of test results, which was proven by Schlechtweg in his study^8^.

When analyzing the above-mentioned data, it should be noted that radiology applications available on mobile devices have a niche, specialized application and do not reach a wide audience. One can come to this conclusion by analyzing the number of installations of individual applications, which for most of them is less than a thousand—in Google Play store this applies to 130 out of 228 applications qualified for this study. For comparison—in the Google Play store, only in September 2022, the TikTok® application was downloaded 23.96 million times and the Instagram application 21.17 million times^13^. In our work we have shown that the fewest applications are related to clinical practice, whereas most are used for educational purposes. This may be the result of their non-admission for clinical use due to the lack of appropriate permissions and certificates. Before a given application is approved for clinical use, it must be first approved by the relevant authorities of a given country or by another superior organization—in the case of the European Union, this is the European Medicines Agency (EMA)^14^. In the United States, the authority issuing these permits is the Food and Drug Administration agency (FDA)^15^. The first mobile application for viewing images and making diagnoses on MRI, X-ray, ultrasound and other radiographic scans for clinical use was approved by the FDA only in 2011^16^.

However, we are satisfied with the large number of educational applications in both stores—154 in the Google Play store and 104 in the App Store. Educational applications can be a handy source of knowledge, available everywhere and at any time, both for students and practicing doctors^17^. De Oliveira et al. present a study on the learning of endodontics with the use of radiographs of various endodontic conditions and emphasize the fact that it positively affects both the learning process (students achieved better results in learning) and diagnosis^18^. Another study conclusively showed that the ability to remember radiology lectures is greater with the use of a remote response device. Millor et al. showed that during learning with an interactive device the number of correct answers was significantly higher than during passive education (63.6 vs. 53.2%, *p* < 0.05). The same applies to the percentage of incorrect answers, which was lower for students using the remote response device (respectively 23,4 vs. 27,4% *p* < 0,005 and 13 vs. 19,5%, *p* < 0,005)^19^. However, the application is not always able to replace traditional teaching methods. In a study by Nilsson et al. the educational achievements of two groups learning ultrasound were compared—one using the application and one using a manual. It has been shown that the use of the application while learning ultrasound does not affect the acquisition of knowledge. There were no statistical differences between the two groups, using an objective scale for assessing the ability to use ultrasound, and there was no significant difference in diagnostic accuracy. The first group which used an app for learning got an average OSAUS (Objective Structured Assessment of Ultrasound Skills scale) score of 42.3% [95% CI 38.5–46.0%], and the other group using only books scored mean-OSAUS of 45.3% [95% CI 39.3–51.3%] (d.f. [1.33] = 0.45, *p* = 0.41)^20^.

The use of mobile applications for consultation and remote diagnosis, especially in emergency situations, is helpful and can shorten the diagnosing time^21,22^. Hidlay et al. showed that when using a smartphone in head and neck diagnostics, in a sample of 76 CT examinations, it provided almost 100% diagnostic accuracy (*p* = 0.9999) using both the PACS workstation and the iPhone®^23,24^. Handelman et al. showed little difference between identifying emergency radiographs on a smartphone via WhatsApp® (accuracy 79,6% ± 7,9%, 11,5%—false positive results, 8,9%—false negative results) and a diagnostic station (accuracy 83,4 ± 6,5%, 8,9%—false positive results, 7,7%—false negative results), which was 6%. However, authors conclude that such a difference does not disqualify the use of WhatsApp as a helpful application for consulting radiographs^25^. WhatsApp also proved to be useful in remote diagnosis of tibial fractures. Demaerschalk et al. showed that the results of the assessment of vascular neurologists using ResolutionMD® on smartphones did not differ from the interpretation of radiologists diagnosing in the traditional way and independent arbiters viewing the images remotely^26^. Thanks to a mobile application implemented in South Korea, radiologists gained greater access to Korean imaging guidelines^27^. Park et al. and Zimmerman et al. showed little difference between coronary artery CT diagnoses made remotely using smartphones or iPad (smartphone: κ = 0.59 and κ = 0.72 for severe stenosis; iPad®: κ = 0.62, 95% CI 0.45–0.79) and comparing with workstation diagnosis (κ = 0.64, 95% CI 0.47–0.81)^28,29^.

Most applications are free—75.08% of App Store offers and 91.23% of Google Play store offers, which makes them easily accessible, regardless of the financial capabilities of users. According to a study by Biviji et al. applications with a higher percentage of downloads were those which were cheaper, were free applications with the option of purchasing individual options already in the application, were applications with a high user rating or were applications in which experts were involved in the creation and development^30^. A large part of the application has been updated recently (in 2021 or 2022)—73 App Store offers and 94 Google Play store offers, so it can be assumed that the knowledge contained therein is up-to-date. Users emphasized the importance of customer service responsiveness, speed in solving problems and meeting their requests for additional features^31^.

In addition, applications containing a reliable description as well as a dedicated website and photos or a video, showing the application's functions, will probably be more willingly chosen by users. Lau et al. proved that users value the visual aspect and nice design of applications more than evidence-based components^32^. Jiang et al. showed that users attach the greatest importance to the description of the application when choosing the application they intend to download. A high-quality description is one that briefly lists and describes the app's features, includes developer contact information and information about permission and requirement^33^.

## Conclusion

Clinical applications could help radiologists in their daily work. First, however, they should be analyzed in order to obtain the appropriate certificate, so that they can be legally used during the healing process. Organizations responsible for issuing the required certificates should pay particular attention to the possibility of using medical applications in clinical practice. Moreover, applications that received "A" category may in our opinion be a good source of knowledge for students of medical faculties or young adepts of radiology. However, we do not rule out applications placed in other categories as long as the knowledge contained in them is up-to-date and presented in an accessible way, they also fulfill their educational role.

## Limitations

We encountered several limitations that effectively prevented us from comparing apps from both stores in a homogeneous way. Limited data availability, mainly the lack of the number of downloads of a given application in the App Store, made it impossible to collect enough information about applications to apply the same application evaluation scheme that was used when assessing applications from Google Play store. Therefore, the rating according to the classification from "A" to "F" was applied only for applications from Google Play store.

Moreover, our article is a pilot study, and mobile applications were assessed subjectively, because due to missing data it was not possible to create a standardized assessment tool. The lack of data availability also made statistical analysis difficult.

Perhaps in the future, with further analysis and greater data availability, a more objective tool can be created and the assessment can be re-evaluated.

## Data Availability

Search results are available from Jakub Kufel.
